# Supplementary material for: Comparative multiomics analysis of cell physiological state after culture in a basket bioreactor
Source: Sci Rep. 2022 Nov 23;12:20161. doi: 10.1038/s41598-022-24687-4 (PMC9686226; doi:10.1038/s41598-022-24687-4)
Supplement: Supplementary file 1 — Supplementary Information 1. [file 41598_2022_24687_MOESM1_ESM.zip › raw data/Proteomic raw data/6.KEGGenrichment/EG.vs.CG/ALL/pathway/src/04621.html]

map04621


- K20909

- Down regulated proteins

A0A0D9QWT4(Description:Anthrax toxin receptor;Gene:ANTXR2;log2FC:-1.7632)

A0A0D9RR35(Description:VWFA domain-containing protein;Gene:ANTXR1;log2FC:-2.1330)

- K20897, K20898

- Down regulated proteins

A0A0D9S6T5(Description:GB1/RHD3-type G domain-containing protein;Gene:;log2FC:-1.1074)

- K20897, K20898, K20899

- Down regulated proteins

A0A0D9S6T5(Description:GB1/RHD3-type G domain-containing protein;Gene:;log2FC:-1.1074)

- K11859

- Down regulated proteins

A0A0D9RWK4(Description:Ubiquitinyl hydrolase 1;Gene:TNFAIP3;log2FC:-1.0404)

Close
